# Supplementary material for: Swinging lever mechanism of myosin directly shown by time-resolved cryo-EM
Source: Nature. 2025 Apr 9;642(8067):519–26. doi: 10.1038/s41586-025-08876-5 (PMC12158783; doi:10.1038/s41586-025-08876-5)
Supplement: Supplementary file 1 — Supplementary Figs. 1–7. [file 41586_2025_8876_MOESM1_ESM.pdf]

nature portfolio

https://doi.org/10.1038/s41586-025-08876-5

Supplementary information

Swinging lever mechanism of myosin directly shown by time-resolved cryo-EM

In the format provided by the authors and unedited

Manuscript 2024-01-00287C
Klebl et al. Swinging lever mechanism of myosin directly shown by time-resolved cryoEM

Supplementary Information

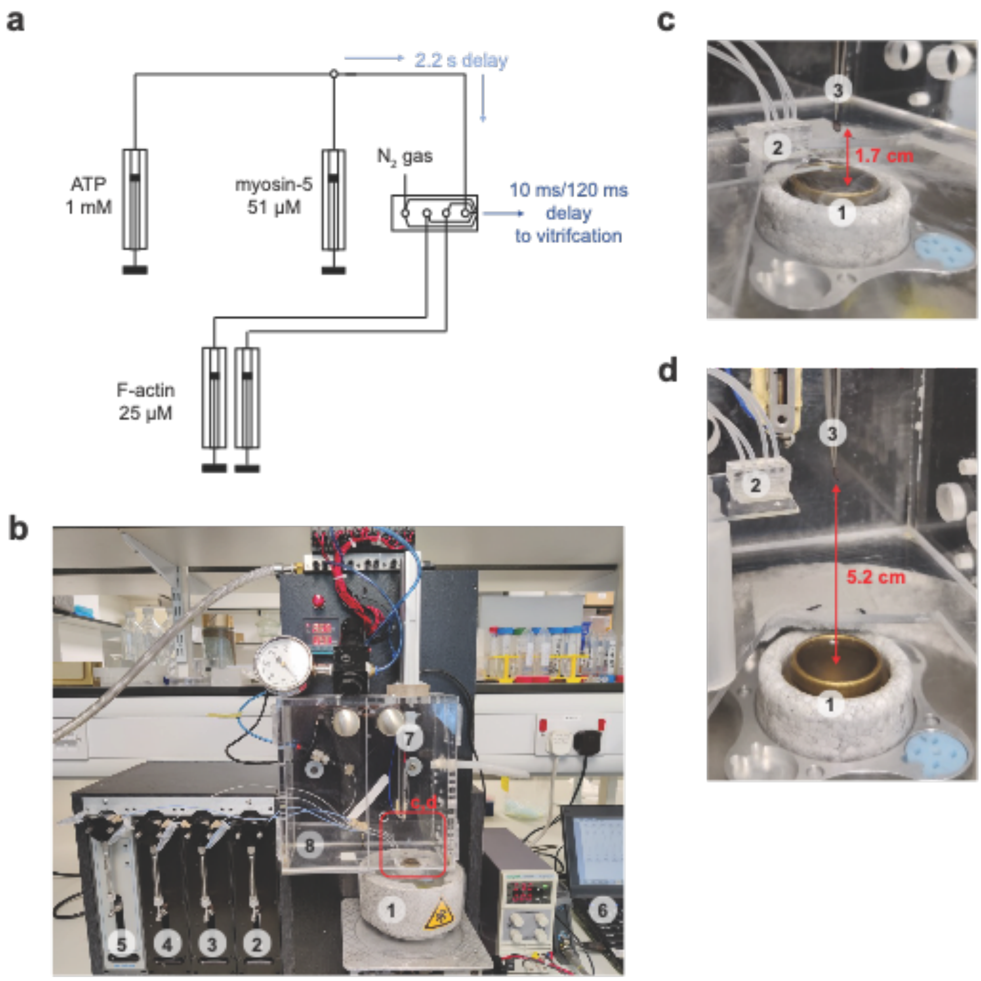

Supplementary Fig 1. Experimental setup for time-resolved cryoEM. (a) Schematic of experimental setup showing the concentrations of reagents used. (b) Photo of the experimental setup with liquid nitrogen/ethane container (1), syringe pumps (2-5), control PC (6), forceps on plunger (7) and humidity controlled chamber (8). The red box highlights the region around the spray nozzle, magnified views of this region are shown in c-d. (c) Magnified view of ethane cup (1), spray nozzle (2) and grid in sample application position (3) with a short distance for the 10 ms timepoint. (d) Similar to c, except for the larger distance between nozzle and ethane that was used for the 120 ms timepoint.

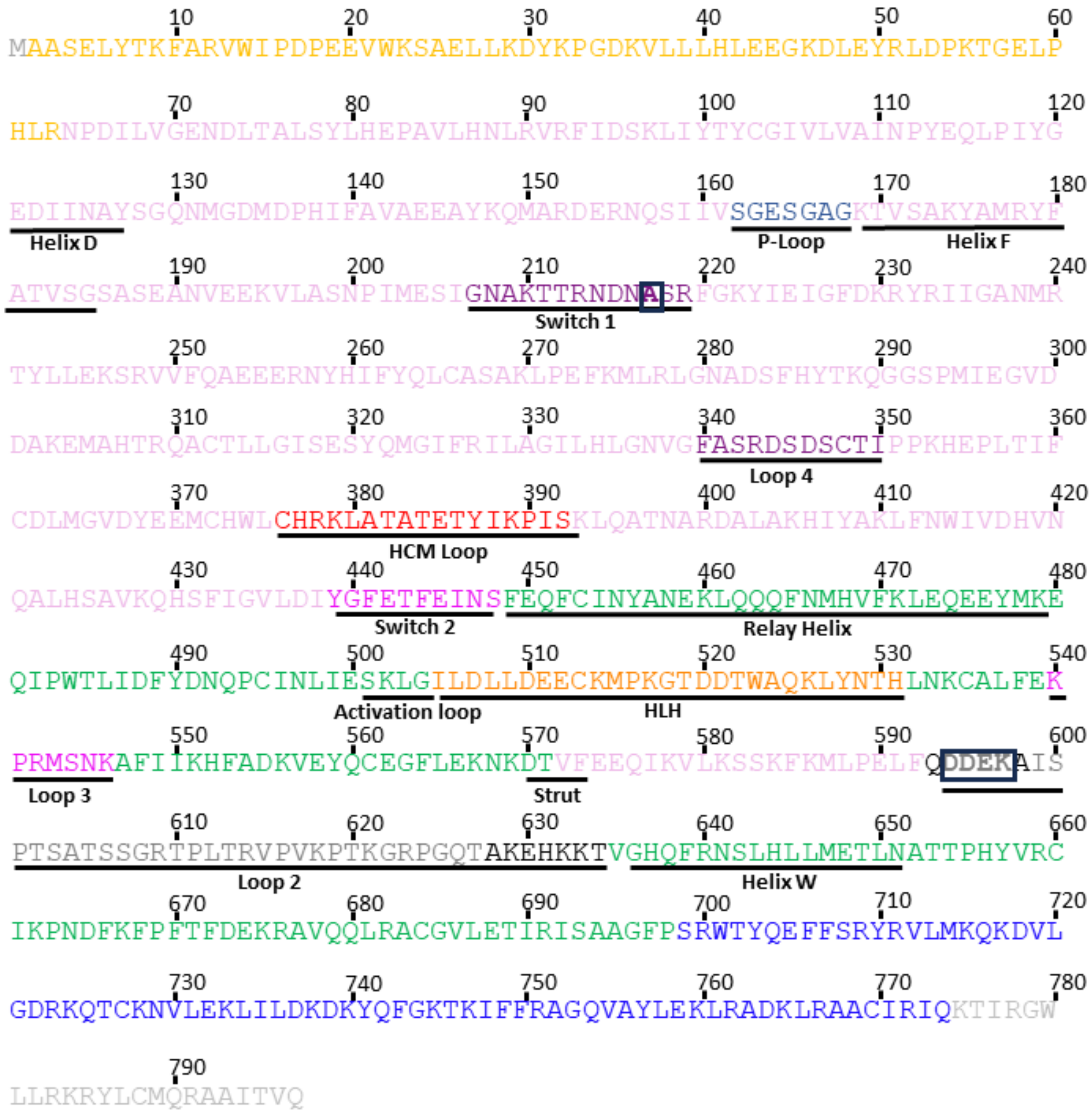

Supplementary Fig. 2. Myosin S1 sequence and domain architecture. Myosin-5 S1 amino acid sequence (myosin heavy chain residues 1-797). Subdomains and regions of interest are colored as in Fig.1-4 and underlined. Gold, N-terminal domain; pink, U50; navy blue, P-loop; purple, switch-1 and loop4; red, HCM loop; magenta, switch-2 and loop3; green, L50; orange, HLH; black/grey, loop2, where residues in black are modelled in our primed actomyosin structure and those in grey are not; royal blue, converter (residues 699-765) and modelled region of light chain binding domain; light grey residues 775-797 are the unmodeled region of the light chain binding domain of the construct. The switch 1 $S^{217}A$ mutation and loop2 $DDEK^{594\text{-}597}$ deletion are boxed. The construct studied has a FLAG-tag sequence, DYKDDDDK, immediately C-terminal to the myosin heavy chain sequence shown.

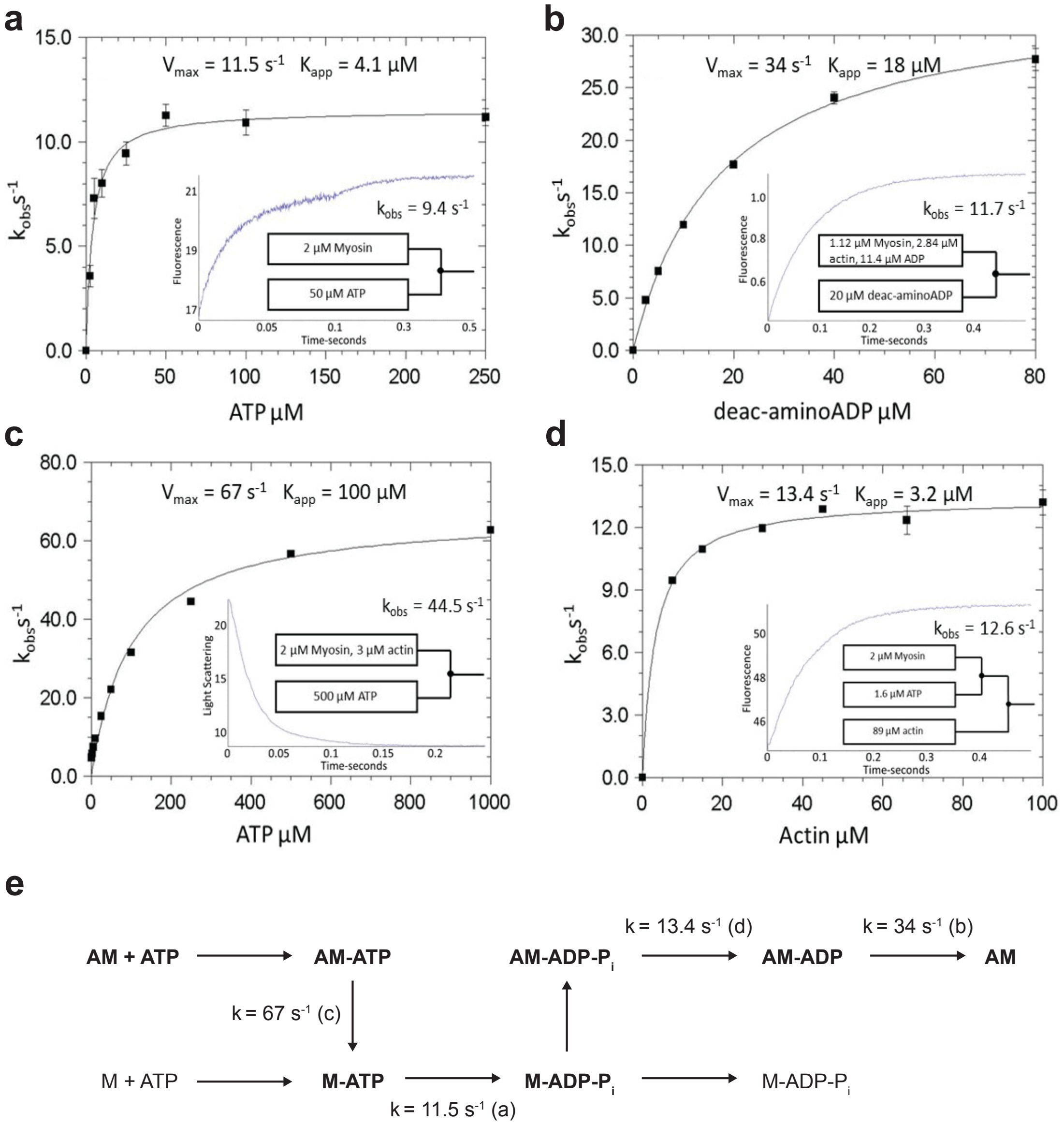

Supplementary Fig. 3. Transient kinetics of mutant actomyosin-5 ATPase. Kinetics measured at 20 °C by single mixing (a-c) or double mixing (d) stopped-flow. Final concentrations in the cell: 37.5 mM KAc, 25 mM KCl, 10 mM MOPS (pH 7.0), 2.25 mM $MgCl_2$, 0.1 mM EGTA, 0.25 mM DTT. Representative traces shown in insets with a stopped-flow mixing schematic. Note protein concentrations stated are those within the syringes, prior to mixing, rather than in the final reaction mixture. **(a)** Myosin ATP hydrolysis measured by intrinsic tryptophan fluorescence using a 320-380 nm bandpass filter with excitation at 295 nm. Final concentrations: 1.0 µM myosin, 1.5 µM calmodulin, and 2.5 - 250 µM ATP. The hyperbolic fit yields V_{max} = 11.5 s^{-1}, K_{app} = 4.1 µM. **(b)** ADP dissociation from actomyosin-ADP was measured using a deac-aminoADP chase with a 455 nm long-pass filter and excitation at 430 nm. Final concentrations: 0.14 µM or 0.56 µM myosin and calmodulin, 1.42 µM actin, 5.7 µM ADP, and 2.5 - 80 µM deac-aminoADP. The hyperbolic fit yields V_{max} = 34 s^{-1}, K_{app} = 18 µM. **(c)** ATP-induced dissociation of myosin from actin measured by light scattering with a 400 nm long-pass filter and illumination at 432 nm. Final concentrations: 1 µM myosin, 1 µM calmodulin, 1.5 µM actin, and 1 - 1000 µM ATP. The hyperbolic fit yields V_{max} = 67 s^{-1}, K_{app} = 102 µM. **(d)** Phosphate dissociation from the actomyosin-ADP-Pi complex, measured by MDCC-PBP

with a 455 nm long-pass filter and excitation at 434 nm. 2 μM myosin was mixed with 1.6 μM ATP, held in a delay line for 2 s, and then mixed with an equal volume of actin to accelerate P_i release. Final concentrations: 0.5 μM myosin, 0.5 μM calmodulin, 0.4 μM ATP, 0 - 100 μM actin, 5 μM MDCC-PBP, 0.1 mM 7-methylguanosine, and 0.01 unit/mL purine nucleoside phosphorylase. The hyperbolic fit yields V_{max} = 13.4 s^{-1}, K_{app} = 3.2 μM. (**e**) Kinetic mechanism of mutant actomyosin-5 S1 ATPase. Abbreviations: A, actin; M, myosin-5 S1(1IQ, S217A, ΔDDEK$^{594-597}$); Pi, phosphate. The main actomyosin ATPase pathway is in bold. Parentheses indicate from which experiment the rates were obtained. Experiments were repeated in triplicate (n = 3 independent replicates). Error bars represent standard deviation from the mean.

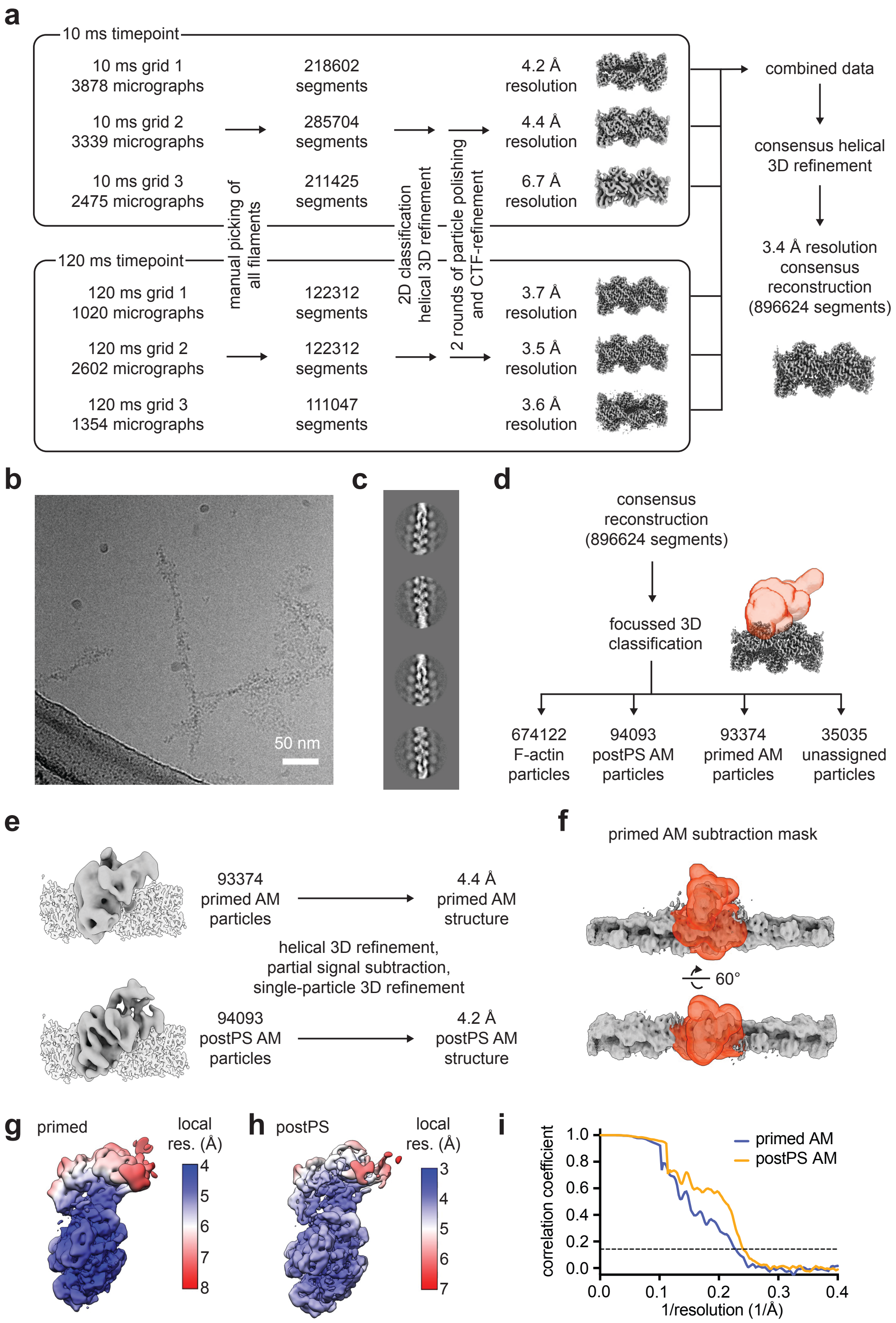

Supplementary Fig. 4. Processing of time-resolved cryoEM data. (a) Flow diagram of the initial image processing of the 10 ms and 120 ms data sets. (b) Micrograph from the 10 ms dataset. (c) 2D classes from the 10 ms timepoint, bound myosin appears as a diffuse density along the actin filament. (d) Result of the focused 3D classification of the combined dataset with a mask covering the myosin binding site (AM: actomyosin). (e) Processing of primed or postPS actomyosin after focused classification. (f) Subtraction mask used for primed actomyosin processing. (g) Final primed actomyosin reconstruction showing local resolution in Å. (h) Final postPS actomyosin reconstruction showing local resolution in Å (i) Fourier shell correlation curves for primed (blue) and postPS (yellow) with the 0.143 threshold indicated by a dotted line.

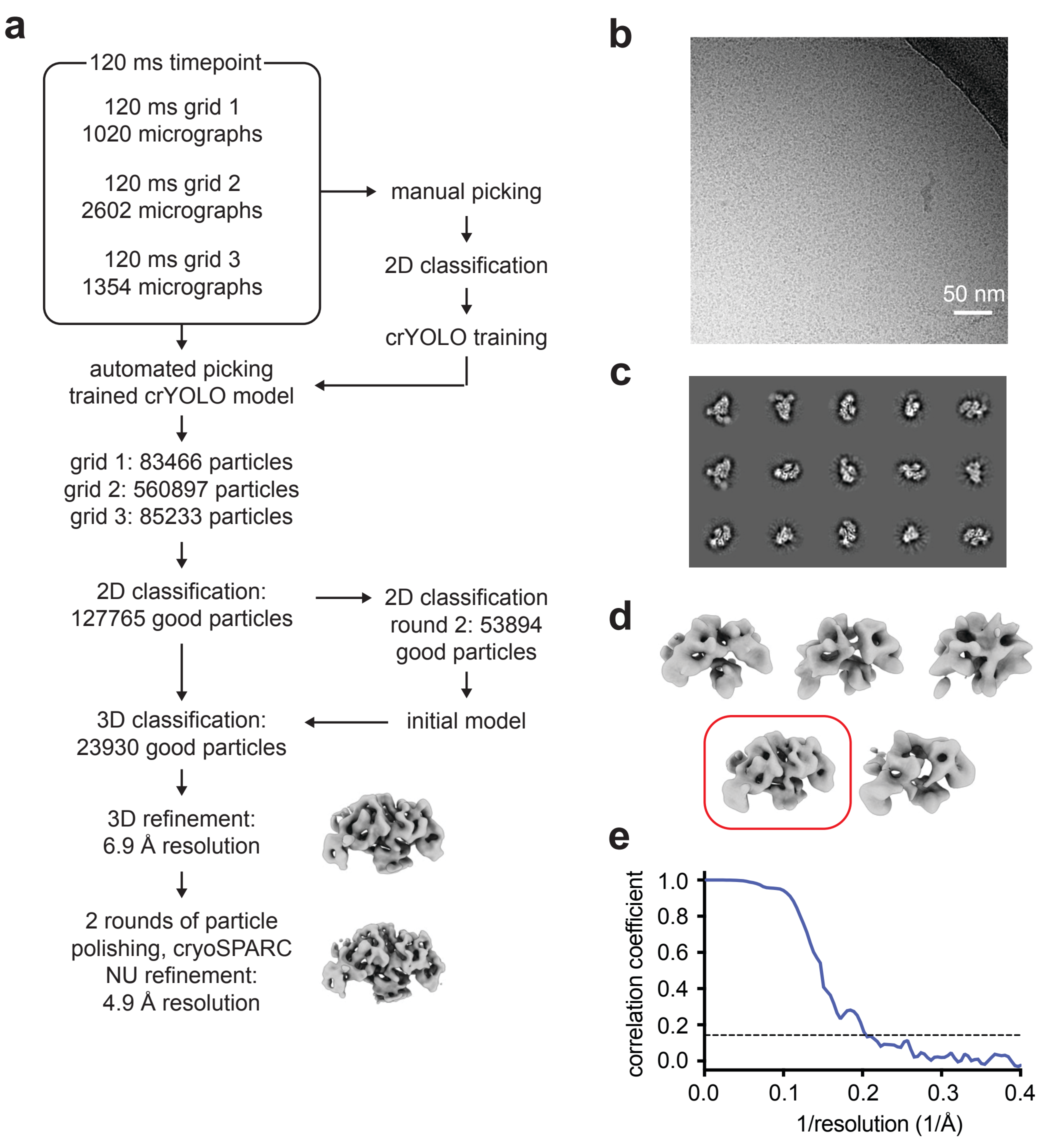

Supplementary Fig. 5. Unbound myosin-5 image processing. (a) Processing pipeline for unbound myosin molecules. (b) Micrograph from the 120 ms time-resolved cryoEM data with a large number of unbound myosin-5 molecules. (c) Representative 2D classes. (d) 3D classification with the selected class highlighted by a red box. (e) Fourier shell correlation curve (blue) with the 0.143 threshold indicated by a dotted line.

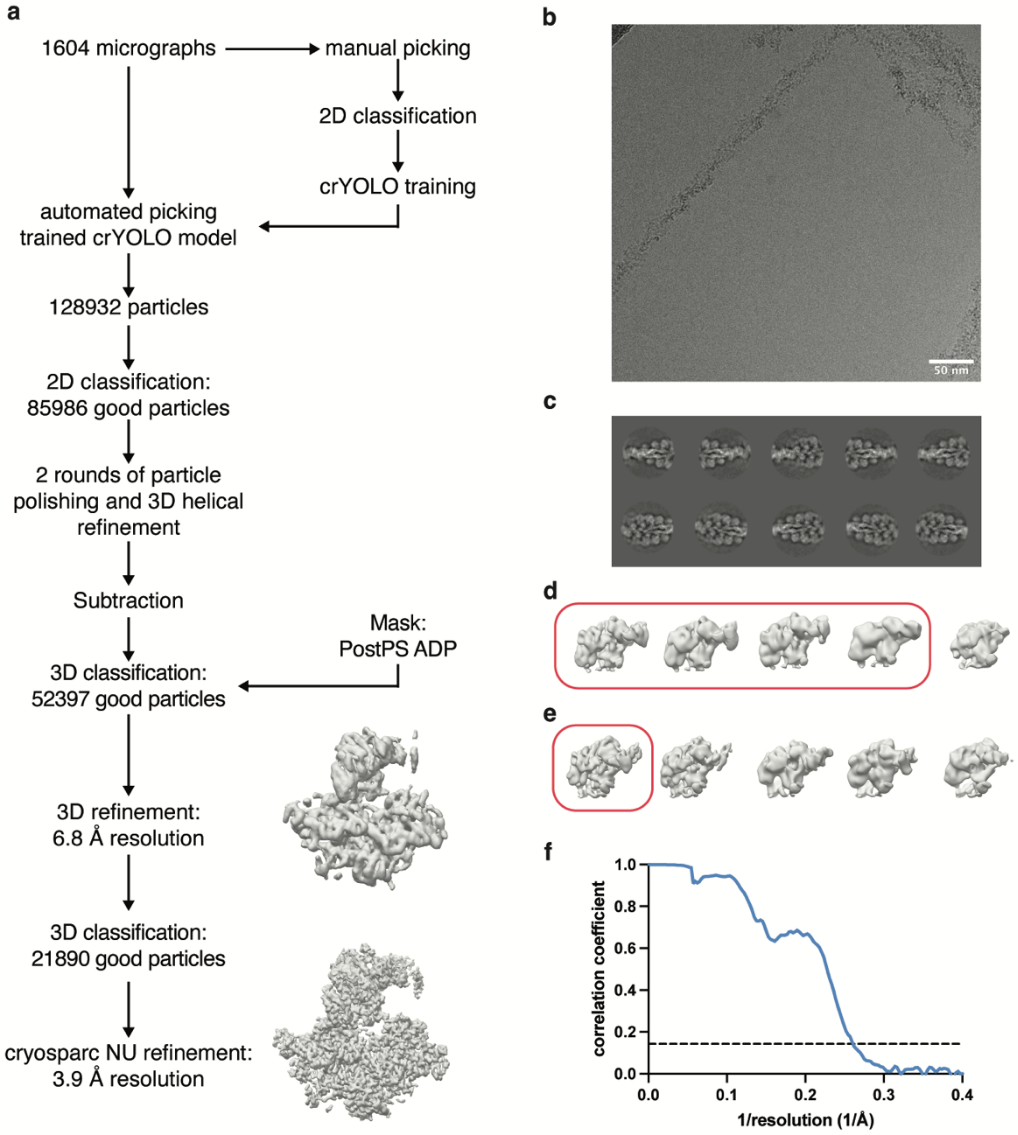

Supplementary Fig. 6. Rigor actomyosin-5 image processing. (a) Processing pipeline. (b) Representative micrograph from the rigor actomyosin-5 dataset. (c) Representative 2D classes. (d) First round of 3D classification with the classes selected for further processing highlighted by a red box. (e) Second round of 3D classification with the class selected for further processing (CRYOSPARC non-uniform refinement) highlighted by a red box. (f) Fourier-shell correlation curve (blue) with the 0.143 threshold indicated by a dotted line.

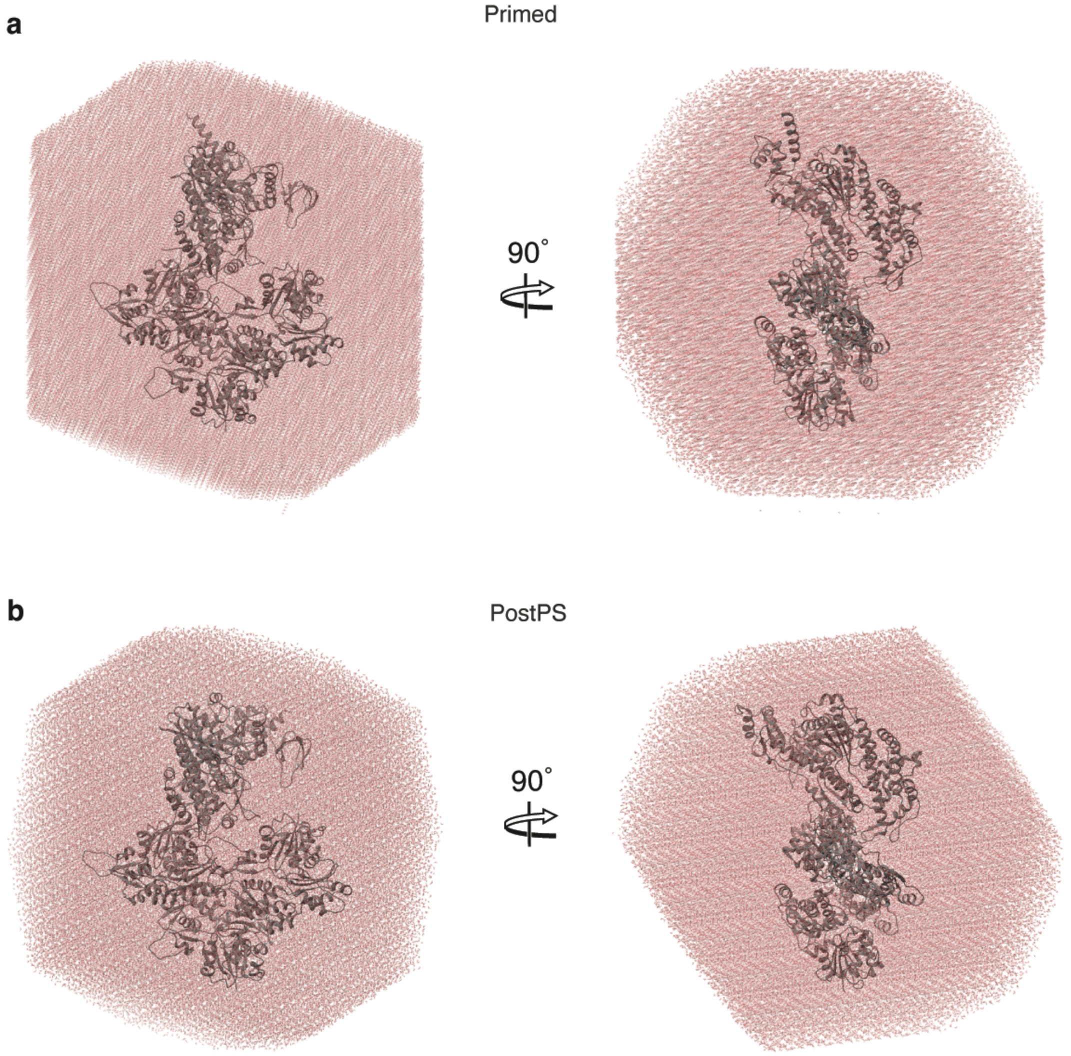

Supplementary Fig. 7. Molecular dynamics simulation solvent box. Octahedral water box used during molecular dynamics simulation. (a) Primed actomyosin and (b) postPS actomyosin models shown in grey with TIP3P water coloured in pink.
