# Supplementary material for: Swinging lever mechanism of myosin directly shown by time-resolved cryo-EM
Source: Nature. 2025 Apr 9;642(8067):519–26. doi: 10.1038/s41586-025-08876-5 (PMC12158783; doi:10.1038/s41586-025-08876-5)
Supplement: Supplementary file 2 — Reporting Summary [file 41586_2025_8876_MOESM2_ESM.pdf]

nature portfolio

Corresponding author(s): Charlotte Scarff, Stephen Muench, Howard White

Last updated by author(s): Feb 10, 2025

Reporting Summary

Nature Portfolio wishes to improve the reproducibility of the work that we publish. This form provides structure for consistency and transparency in reporting. For further information on Nature Portfolio policies, see our Editorial Policies and the Editorial Policy Checklist.

Statistics

For all statistical analyses, confirm that the following items are present in the figure legend, table legend, main text, or Methods section.

n/a	Confirmed	
☐	☒	The exact sample size (*n*) for each experimental group/condition, given as a discrete number and unit of measurement
☐	☒	A statement on whether measurements were taken from distinct samples or whether the same sample was measured repeatedly
☒	☐	The statistical test(s) used AND whether they are one- or two-sided<br>*Only common tests should be described solely by name; describe more complex techniques in the Methods section.*
☒	☐	A description of all covariates tested
☒	☐	A description of any assumptions or corrections, such as tests of normality and adjustment for multiple comparisons
☐	☒	A full description of the statistical parameters including central tendency (e.g. means) or other basic estimates (e.g. regression coefficient) AND variation (e.g. standard deviation) or associated estimates of uncertainty (e.g. confidence intervals)
☒	☐	For null hypothesis testing, the test statistic (e.g. *F*, *t*, *r*) with confidence intervals, effect sizes, degrees of freedom and *P* value noted<br>*Give P values as exact values whenever suitable.*
☐	☒	For Bayesian analysis, information on the choice of priors and Markov chain Monte Carlo settings
☒	☐	For hierarchical and complex designs, identification of the appropriate level for tests and full reporting of outcomes
☒	☐	Estimates of effect sizes (e.g. Cohen's *d*, Pearson's *r*), indicating how they were calculated

Our web collection on statistics for biologists contains articles on many of the points above.

Software and code

Policy information about availability of computer code

Data collection	Data were collected on a Titan Krios microscope equipped with a Gatan K2 direct electron detector operated in counting mode using the EPU software version 2.12 distributed by Thermo Scientific.
Data analysis	Data processing was done using RELION-3.1 and cryoSPARC version 3.2.0. Micrographs were corrected for beam-induced motion using MotionCor2 and CTF estimation was done using GCTF. Post-processing was performed in RELION and in DeepEMhancer. Homology models were generated using Modeller within Chimera. Model building was done using coot, with subsequent refinement of the nucleotide pocket in ISOLDE. Real space refinement was performed using Phenix. Videos were generated by use of Chimera, Adobe Aftereffects and Adobe Premiere. All simulations were run in AMBER16 (GPU version PMEMD-CUDA) on all atom systems parameterised with the charmm36m forcefield and built using CHARMM-GUI. The primed and postPS actomyosin structures were prepared for molecular dynamics simulations using the CHARMM-GUI solution builder.

For manuscripts utilizing custom algorithms or software that are central to the research but not yet described in published literature, software must be made available to editors and reviewers. We strongly encourage code deposition in a community repository (e.g. GitHub). See the Nature Portfolio guidelines for submitting code & software for further information.

Data

Policy information about availability of data

All manuscripts must include a data availability statement. This statement should provide the following information, where applicable:

- Accession codes, unique identifiers, or web links for publicly available datasets
- A description of any restrictions on data availability
- For clinical datasets or third party data, please ensure that the statement adheres to our policy

The electron density maps and atomic models for unbound primed myosin-5, primed actomyosin-5 and postPS actomyosin-5 have been deposited into EMDB, with accession codes EMD-19031, EMD-19013 and EMD-19030, and the PDB with accession codes 8RBG, 8R9V and 8RBF, respectively. The electron density map for rigor actomyosin-5 has been deposited into the EMDB with accession code EMD-50594.
The following models were used for comparison purposes in our study, actomyosin-5 rigor structures PDB IDs: 7PLT, 7PLU, 7PLV, 7PLW, 7PLZ and actomyosin-5 strong-ADP structures PDB IDs: 7PM5, 7PM6, 7PM7, 7PM8, 7PM9.
Each MD trajectory and parameter-topology file for primed and PostPS structures for each MD simulation replicate is provided along with the starting models at: https://doi.org/10.6084/m9.figshare.24948180

Research involving human participants, their data, or biological material

Policy information about studies with human participants or human data. See also policy information about sex, gender (identity/presentation), and sexual orientation and race, ethnicity and racism.

Reporting on sex and gender	N/A
Reporting on race, ethnicity, or other socially relevant groupings	N/A
Population characteristics	N/A
Recruitment	N/A
Ethics oversight	N/A

Note that full information on the approval of the study protocol must also be provided in the manuscript.

Field-specific reporting

Please select the one below that is the best fit for your research. If you are not sure, read the appropriate sections before making your selection.

☒ Life sciences ☐ Behavioural & social sciences ☐ Ecological, evolutionary & environmental sciences

For a reference copy of the document with all sections, see nature.com/documents/nr-reporting-summary-flat.pdf

Life sciences study design

All studies must disclose on these points even when the disclosure is negative.

Sample size	Sample size was determined based on the required particle number to achieve the desired resolution
Data exclusions	Data were excluded during the 2D and 3D classification stage based on typical data processing procedures as shown in image processing figures
Replication	Time points (10 ms and 120 ms respectively) were repeated 3 times as described in the manuscript to show reproducibility
Randomization	Data processing approaches used (maximum likelihood classification) ensure randomization and unbiased particle classification
Blinding	Blinding was not required. Whilst different datasets were acquired at time points of 10 and 120 ms respectively these were combined and analyzed by the same methods.

Reporting for specific materials, systems and methods

We require information from authors about some types of materials, experimental systems and methods used in many studies. Here, indicate whether each material, system or method listed is relevant to your study. If you are not sure if a list item applies to your research, read the appropriate section before selecting a response.

Materials & experimental systems

n/a	Involved in the study	
☒	☐	Antibodies
☒	☐	Eukaryotic cell lines
☒	☐	Palaeontology and archaeology
☒	☐	Animals and other organisms
☒	☐	Clinical data
☒	☐	Dual use research of concern
☒	☐	Plants

Methods

n/a	Involved in the study	
☒	☐	ChIP-seq
☒	☐	Flow cytometry
☒	☐	MRI-based neuroimaging

Plants

Seed stocks	N/A
Novel plant genotypes	N/A
Authentication	N/A
